# Clinical Neuropathology Practice Guide 3-2013: levels of evidence and clinical utility of prognostic and predictive candidate brain tumor biomarkers 

**DOI:** 10.5414/NP300646

**Published:** 2013-05-02

**Authors:** Anna S. Berghoff, Harald Stefanits, Adelheid Woehrer, Harald Heinzl, Matthias Preusser, Johannes A. Hainfellner

**Affiliations:** 1 Institute of Neurology,; 2 Department of Neurosurgery,; 3 Center for Medical Statistics, Informatics, and Intelligent Systems,; 4 Department of Medicine I,; 5 Comprehensive Cancer Center CNS Unit, Medical University of Vienna, Vienna, Austria

**Keywords:** biomarker, brain tumor, neurooncology, prognostic marker, predictive marker, analytical performance, clinical performance, clinical utility

## Abstract

A large number of potential tissue biomarkers has been proposed for brain tumors. However, hardly any have been adopted for routine clinical use, so far. For most candidate biomarkers substantial controversy exists with regard to their usefulness in clinical practice. The multidisciplinary neurooncology taskforce of the Vienna Comprehensive Cancer Center Central Nervous System Unit (CCC-CNS) addressed this issue and elaborated a four-tiered levels-of-evidence system for assessing analytical performance (reliability of test result) and clinical performance (prognostic or predictive) based on consensually defined criteria. The taskforce also consensually agreed that only biomarker candidates should be considered as ready for clinical use, which meet defined quality standards for both, analytical and clinical performance. Applying this levels-of-evidence system to *MGMT*, *IDH1*, 1p19q, Ki67, *MYCC*, *MYCN* and β-catenin, only immunohistochemical *IDH1* mutation testing in patients with diffuse gliomas is supported by sufficient evidence in order to be unequivocally qualified for clinical use. For the other candidate biomarkers lack of published evidence of sufficiently high analytical test performance and, in some cases, also of clinical performance limits evidence-based confirmation of their clinical utility. For most of the markers, no common standard of laboratory testing exists. We conclude that, at present, there is a strong need for studies that specifically address the analytical performance of candidate brain tumor biomarkers. In addition, standardization of laboratory testing is needed. We aim to regularly challenge and update the present classification in order to systematically clarify the current translational status of candidate brain tumor biomarkers and to identify specific research needs for accelerating the translational pace.

## Introduction 

In clinical medicine biomarkers are defined as objectively measurable/determinable patient-related factors that provide clinically meaningful disease-related information with regard to diagnosis, prognosis, therapy decisions and patient follow-up [1, 2, 3]. In neuropathological oncology, diagnostic, prognostic and predictive biomarkers assessed in patient biopsy specimens and/or body fluids are of relevance [4, 5]. A large number of prognostic and predictive candidate tissue biomarkers have been proposed for brain tumors, but almost none have translated into routine clinical use so far [4, 6]. For most biomarkers, there is substantial controversy regarding their clinical usefulness [5]. In this article, we present a levels-of-evidence system for assessing the current translational status of candidate biomarkers. This levels-of-evidence system is based on criteria, which have been elaborated and consensually defined by the multidisciplinary neurooncology task force of the Vienna Comprehensive Cancer Center – Central Nervous System Unit (CCC-CNS). We apply this system to currently debated prognostic and predictive neuro-oncological candidate biomarkers in order to assess their clinical utility. 

## Methods and definitions 

Our multidisciplinary neurooncology task force within the CCC-CNS has defined the criteria for a four-tiered levels-of-evidence system and an adjunct scoring system for assessing the clinical utility of prognostic and predictive candidate brain tumor biomarkers in a continued process of discussion and consensual agreement (Table 1). The levels-of-evidence system is related to the two crucial dimensions analytical and clinical performance, which are considered the essential elements for clinical biomarker translation [4]. Having established the levels-of-evidence system, we used it for assessing the evidence levels and clinical utility for the following candidate brain tumor biomarkers: O6-methylguanine methyl-transferase gene (*MGMT*) promoter methylation status, isocitrate dehydrogenase 1 gene (*IDH1*) mutation status, chromosome arms 1p19q co-deletion status, Ki67 tumor cell proliferation index, *MYCN* status, *MYCC* status and β-catenin expression. We selected these biomarker candidates, because they are considered to be close to routine clinical use, but their translational status is still subject to controversy and discussion. 

For each candidate biomarker, we separately assessed the analytical performance and the prognostic and predictive clinical performance. Our ratings are based on review of published data and rely on consensual agreement within our multidisciplinary task force. 

We defined analytical performance as the reliability of the results yielded by a particular assessment or test. To this end, we evaluated published data with regard to repeatability of test results (intra-laboratory agreement, intra-observer agreement) and reproducibility of testing (inter-laboratory agreement, inter-observer agreement). 

As clinical performance, we defined the prognostic and predictive value of a given candidate biomarker. Prognostic markers were defined by their association with patient outcome, and predictive markers by their association with response to a given therapy. As the definition of prognostic and predictive markers in (neuro)oncology has been subject to continued debate and controversy, we provide – for illustration purpose – a generic example of a biomarker in medicine with prognostic, predictive and diagnostic properties, depending on the issue of interest (see Textbox). We perceive this basic biomarker concept also as valid for the field of neurooncology. 

Only those factors reaching an A or B level for both analytical and cliniical performance were considered to have adequate justification for recommendation in routine clinical use as prognostic or predictive biomarkers (Table 1). 

## Prognostic and predictive candidate biomarkers (see also Table 2) 

## Isocitrate dehydrogenase (IDH) mutation status 

**IDH testing – analytical performance**

A recently published ring trial (round robin test) and several studies including large patient cohorts support the high analytical performance of immunohistochemical detection of the *IDH1-R132H* mutation [7, 8, 9, 10, 11, 12]. In contrast, DNA-based *IDH* sequencing procedures showed inconsistent results among different laboratories [12]. 

**IDH testing – prognostic and predictive clinical performance**

The high prognostic clinical performance of *IDH* mutations in diffuse gliomas was confirmed in several large cohorts [7, 13, 14, 15]. However, all of these studies had a retrospective design. Data from adequately designed prospective trials have not yet been published. With regard to a potential predictive value of *IDH1* mutations, there is currently only little evidence based on few and inconsistent results from small studies [7, 16, 17, 18]. 

**Interpretation of the clinical utility of IDH testing**

Assessment of *IDH1-R132H* mutation status by immunohistochemistry has sufficient evidence for clinical use as a prognostic marker in diffuse gliomas. IDH testing by DNA-based methods, which is in principle suitable to detect also other and rarer forms of IDH mutations, has promising clinical utility but is not yet ready for routine clinical use. Standard test protocols with the potential of sufficiently high analytical performance need to be elaborated and validated. 

## 1p19q co-deletion 

**1p19q testing – analytical performance**

Published data indicate promising analytical performance of the following methods for 1p19q co-deletions testing in oligodendroglial tumors: fluorescence in situ hybridization (FISH), PCR-based loss of heterozygosity (LOH) analysis and multiplex ligation-dependent probe amplification (MLPA). FISH seems to be the most robust method allowing also visual control of test results. However, a published ring trial formally documenting the high inter-laboratory reproducibility is missing [19]. 

**1p19q testing – clinical performance**

There is a high level of evidence for the prognostic value of 1p19q co-deletion in oligodendroglial tumors coming from several studies, including two large prospective trials [14, 20]. These studies also indicate a promising predictive value of the 1p19q co-deletion with regard to a response to PCV (procarbazine, lomustine, vincristine)-based chemotherapy in anaplastic oligodendroglial tumors. 

**1p19q testing – clinical utility**

There is strong evidence that 1p19q co-deletion has a prognostic and predictive value in anaplastic oligodendroglial tumors. Ring trials remain to be performed in order to objectively validate the high reproducibility of 1p19q testing and thus making it recommendable for routine clinical use. 

## Ki67 tumor cell proliferation index 

**Ki67 proliferation index analysis – analytical performance**

Several methods for Ki67 proliferation index analysis exist, such as direct counting, semi quantitative estimation, computer-based image analysis and direct microscope-assisted counting with a graticule. These assessments are associated with relatively high reproducibility among trained observers [21, 22, 23, 24, 25]. However, Ki67 immunostaining procedures lack standardization of antigen retrieval and staining. Modalities of tissue fixation may also impact on Ki67 staining results. This lack of standardization may limit reproducibility among different laboratories [26]. 

**Ki67 tumor cell proliferation index analysis – clinical performance**

A robust association of high Ki67 tumor cell proliferation index with unfavorable survival times in patients with ependymoma has been shown in several independent retrospective series [25, 27, 28, 29]. For non-functioning pituitary adenomas promising data exist to support an inverse correlation of Ki67 tumor cell proliferation index with time to progression [30, 31, 32]. With regard to the prognostic value of Ki67 tumor cell proliferation index, only small studies or conflicting results exist for oligodendroglial tumors, diffuse astrocytomas, meningiomas and medulloblastomas [24, 33, 34, 35, 36, 37, 38, 39, 40, 41, 42, 43, 44]. Several studies indicate that the Ki67 tumor cell proliferation index has no prognostic value in glioblastomas [36, 38, 45]. For none of the mentioned entities sufficient data exist to support a predictive value of the Ki67 tumor cell proliferation index. 

**Ki67 tumor cell proliferation index analysis – clinical utility**

The Ki67 tumor cell proliferation index is associated with a high prognostic clinical performance in ependymoma. However, inter-laboratory variability of tissue processing and immunohistochemical staining protocols limit its clinical utility. There is a need for better standardization of tissue processing and Ki67 immunostaining procedures. 

## MGMT methylation 

**MGMT methylation testing – analytical performance**

Various DNA-based methods for testing of MGMT promoter methylation status have been developed, but for none of them intra- and interlaboratory reproducibility has been sufficiently analyzed [46, 47, 48, 49, 50]. 

**MGMT methylation testing – clinical performance**

The high prognostic value of MGMT methylation in glioblastoma has been confirmed by many studies including prospective trial data [51, 52]. A predictive value of MGMT promoter methylation status for response to temozolomide-based chemotherapy in elderly glioblastoma patients is supported by two independent prospective randomized clinical trials [53, 54]. 

**MGMT methylation testing – clinical utility**

In glioblastoma, a high prognostic clinical performance of MGMT promoter methylation status has been robustly confirmed. In the subgroup of elderly patients there is also evidence for a high predictive value. However, there is insufficient evidence for a high analytical test performance. In particular, intra- and interlaboratory reproducibility remains to be confirmed by adequate scientific data. This lack of evidence impedes recommendation of MGMT testing for routine clinical use. 

## *MYCC* amplification 

**MYCC amplification testing – analytical performance**

Promising data in terms of analytical performance exist for FISH-based analysis in medulloblastoma [55, 56, 57, 58]. Trials analyzing the inter-observer and inter-laboratory reproducibility have not been performed so far, but currently studies are accomplished to test the analytical performance. 

**MYCC amplification testing – clinical performance**

The investigation of the clinical performance of *MYCC* amplification is limited by sample size issues, as medulloblastomas are relatively rare and only ~ 5% of the cases harbour *MYCC* amplification. However, several retrospective studies consistently show an association of poor patient outcome and *MYCC* amplification status [55, 58, 59, 60]. In forthcoming SIOP PNET clinical trials, patients with tumors harboring a *MYCC* amplification will be excluded from the average risk medulloblastoma group and included into the high-risk patient group. 

**MYCC amplification testing – clinical utility**

Data indicate a significant prognostic value of *MYCC* gene amplification status in medulloblastoma. Currently, there is still a lack of data confirming a high analytical performance of *MYCC* amplification testing. 

## *MYCN* amplification 

**MYCN amplification testing – analytical performance**

Like in *MYCC* gene amplification testing, currently available data suggest that FISH has the highest analytical performance for identification of *MYCN* amplifications [55, 56, 57, 58]. No ring trial systematically investigating the inter-observer and inter-laboratory reproducibility has been reported so far. Currently, studies are performed to test the analytical performance. 

**MYCN amplification testing – clinical performance**

Similar as *MYCC* only ~ 5% of medulloblastomas harbor MYCN amplification. A prognostic value of *MYCN* amplification has been reported in several studies [55, 58, 59, 60]. Recent data suggest that *MYCN* amplified medulloblastomas comprise two different molecular subgroups with different clinical characteristics and prognosis [61]. Thus, to date available data do not support a predictive value of *MYCN* gene amplification status in the whole medulloblastoma cohort, and refinement of the definition of *MYCN* amplified tumors by using additional clinical and molecular parameters is needed. In the forthcoming SIOP PNET5/6 clinical trial for average and low-risk medulloblastomas, patients with tumors harboring a *MYCC* amplification will be excluded and treated with a high risk protocol which is currently developed. 

**MYCN amplification testing – clinical utility**

Currently, there is insufficient evidence, both for analytical and clinical performance of *MYCN* gene amplification status testing in medulloblastoma. Therefore, it does not fulfill the criteria for being implemented in the routine clinical setting. There is a need to systematically address both, analytical and clinical performance of *MYCN* amplification testing in adequately designed studies. 

## β-catenin status 

**β-catenin testing – analytical performance**

The β-catenin status in medulloblastomas can be tested by immunohistochemistry alone or in combination with gene sequencing [62, 63, 64, 65]. No ring trials and consensus guidelines for β-catenin immunohistochemistry evaluating the analytical performance of β-catenin testing have been conducted so far. Currently, studies are performed to test the analytical performance of β-catenin immunohistochemistry. 

**β-catenin testing – clinical performance**

β-catenin protein expression and mutations within the β-catenin encoding gene (*CTNNB1*) have been associated with improved patient outcome in several retrospective medulloblastoma studies including large patient cohorts [59, 64, 66, 67], but this correlations have so far not been confirmed in prospective studies. The predictive value of the β-catenin status in medulloblastoma will be tested in the forthcoming SIOP PNET5 clinical trial. 

In supratentorial primitive neuroectodermal tumors (CNS PNET), several independent studies have shown that β-catenin mutation is neither a prognostic nor predictive marker [68]. 

**β-catenin testing – clinical utility**

There is evidence for a prognostic value of β-catenin testing in medulloblastomas. However, there is a need for ring trials and elaboration of consensus guidelines for standardization of laboratory protocols for β-catenin testing. The prognostic value of the β-catenin status in medulloblastoma needs to be confirmed in adequately designed studies. 

## Discussion 

Among the biomarker candidates that were evaluated according to our levels-of-evidence system, only immunohistochemical testing for IDH1-R132H mutations unequivocally meets the criteria that indicate sufficient evidence for routine clinical use. 

The most common and important reason why the other candidate biomarkers failed to meet the criteria for routine clinical use was lacking evidence of sufficiently high analytical test performance. This result indicates that there is a strong need of studies that specifically address the issue of test reproducibility, e.g., by means of repeatability testing, ring trials and interlaboratory comparison, as has been done recently in the case of IDH mutation testing [12, 69]. Indeed, this ring trial can be considered as the final building element definitely translating IDH testing of diffuse gliomas into routine clinical use [69]. 

Another necessity specific to analytical performance is the standardization of testing procedures which constitutes an important prerequisite for interlaboratory comparability of test results. In the case of the widely used Ki67 immunostaining for example, a consensus-based standardized immunohistochemical staining procedure does not exist so far, which limits interlaboratory comparability of Ki67 tumor cell proliferation index threshold values. In the future, Euro-CNS may serve as a common platform for the definition of test standards as has recently been taken place in the case of the 1p/19q FISH protocol [19]. 

The prognostic clinical performance for most of the candidate biomarkers included in this study has been clarified by means of adequately designed and sufficiently powered clinical trials. However, with regard to certain biomarkers relevant for pediatric brain tumors (e.g., *MYCC* and *MYCN* amplification, β-catenin testing), the prognostic value has not yet been sufficiently clarified because of the rarity of these tumors. This limitation underscores the need for sharing of tissue specimens within multicenter research collaborations, in order to increase both the total case numbers and – in further consequence – the statistical power of patient outcome studies. 

For virtually none of the investigated candidate biomarkers, a sufficiently high evidence level for predictive clinical performance exists that would allow rating them as ready for clinical use. The only candidate biomarkers, for which published literature indicates a high predictive clinical performance is MGMT testing in glioblastomas of the elderly and 1p19q status in anaplastic oligodendroglial tumors [14, 20, 51, 52]. However, translation of MGMT testing into common clinical use has been impeded by controversial evidence for a sufficiently high analytical test performance. In order to translate MGMT testing into common clinical use, a robust and reproducible method needs to be established and validated. Validation of currently used MGMT test methods seems to be very challenging, mainly because of methodological reasons [48, 50]. 

## Conclusion 

Our four-tiered levels-of-evidence system allows us to clarify the current translational status and clinical utility of candidate brain tumor biomarkers. Among the currently most debated candidates, only IDH mutation testing in diffuse gliomas is supported by sufficiently high evidence unequivocally qualifying it as prognostic clinical biomarker that is ready for routine clinical use. In the other biomarker candidates, insufficient evidence for high analytical test performance and – in some instances – also low clinical performance (mainly due to low case numbers) seem to be the major obstacles that impede the fast translation of candidate biomarkers into common clinical use. Future studies should be aware of and specifically address these limitations. 

We intend to regularly update the current translational status of candidate brain tumor biomarkers. Such periodic reassessments assessment may also be helpful to identify and exclude inappropriate biomarker candidates, and should be beneficial to identify specific research needs that may help to accelerate the translational pace. 

## Acknowledgments 

We thank Dr. Christine Haberler for helpful discussion and comments. 

## Conflict of interests 

The authors have no conflict of interest to declare. 

**Table 1:** Criteria for the four-tiered CCC-CNS levels of evidence system.

analytical performance	A Robust confirmation of sufficiently high analytical performance by one or more adequately designed and published round robin/ring trials
	B Sufficient evidence for high analytical performance from adequately designed published investigations
	C Promising analytical performance from published investigations
	D Unclear analytical performance based on published single laboratory experience, inconsistent results between different studies, expert opinion
clinical performance (prognostic/predictive)	A Robust confirmation of sufficiently high clinical performance by one or more adequately designed and published prospective studies
	B Sufficient evidence for high clinical performance from adequately designed published investigations
	C Promising clinical performance from published investigations
	D Unclear clinical performance based on published single center experience, inconsistent results between different studies, expert opinion
clinical utility	Both ≥ B = sufficient evidence for routine clinical use At least one < B = insufficient evidence for routine clinical use

**Textbox:** Illustrative generic example of a biomarker in medicine which can be used as prognostic, predictive or diagnostic biomarker, depending on the issue of interest:

*1. Prognostic biomarker:*
Core criterion of a prognostic biomarker: provides information with regard to some outcome over time (e.g., phenotype, survival, etc.).
Proband: child, gender is unknown
Question: as this child grows up, will it adopt a male or female phenotype?
Biomarker: sex chromosomal status from karyogram
Result of biomarker analysis: XY sex chromosomal status
Outcome: when this child becomes an adult, it will adopt a male phenotype, because it is biologically male
This example illustrates that a prognostic marker allows to foresee the result of a natural development over time.
*2. Predictive biomarker:*
Core criterion of a predictive biomarker: provides information whether a particular intervention or therapy is likely to be effective in the tested person or not.
Proband: young adult person of unknown gender
Question: will the administration of oral contraceptives be effective?
Biomarker: sex chromosomal status from karyogram
Result of biomarker analysis: XX sex chromosomal status
Outcome: administration of oral contraceptives will be effective, because the person is biologically female
This example illustrates that a predictive biomarker allows foreseeing the effect of a particular intervention depending on the status of the biomarker.
*3. Diagnostic biomarker:*
Core criterion of a diagnostic biomarker: identifies / confirms a particular entity.
Proband: person, gender is unknown
Question: is this person biologically female or male?
Biomarker: sex chromosomal status from karyogram
Result of biomarker analysis: XY sex chromosomal status
Interpretation: This person is biologically male
This example illustrates that a diagnostic biomarker identifies/confirms a particular entity.

**Table 2:** Candidate biomarkers and their levels of evidence for analytical performance and prognostic/predictive clinical performance.

Biomarker	Studied entities	Studied methods	Analytical performance	Prognostic clinical performance	Predictive clinical performance	Reference
*IDH* mutation	diffuse gliomas	IHC, DNA-based methods	A: R132H-IHC B: C: DNA-based methods D:	A: B: diffuse gliomas C: D:	A: B: C: D: diffuse gliomas	[7, 8, 9, 10, 11, 12, 13, 14, 15, 16, 17, 18, 69]
1p19q co-deletion	oligodendroglial tumors, anaplastic oligodendroglial tumors	FISH, MLPA, LOH	A: B: C: FISH, MLPA, LOH D:	A: oligodendroglial tumors B: C: D:	A: B: C: anaplastic oligodendroglial tumors D:	[14, 19, 20]
Ki 67 proliferation index	ependymoma, glioblastoma, oligodendroglioma, astrocytoma, oligoastrocytom, anaplastic oligodendromglioma, analplastic astrocytoma, anaplastic oligoastrocytoma, meningioma, medulloblastoma, pituitary adenomas (non-functioning)	IHC	A: B: C: IHC D:	A: B: ependymoma, C: pituitary adenomas (non functioning) D: oligodendroglioma, diffuse astrocytoma, meningioma, medulloblastoma	A: B: C: D: ependymoma, glioblastoma, oligodendroglioma, astrocytoma, oligoastrocytoma, anaplastic oligodendroglioma, analplastic astrocytoma, anaplastic oligoastrocytoma, meningioma, medulloblastoma, pituitary adenomas (non-functioning)	[21, 22, 23, 24, 25, 26, 27, 28, 29, 30, 31, 32, 33, 34, 35, 36, 37, 38, 39, 40, 41, 42, 43, 44, 45]
*MGMT* methylation	glioblastoma	DNA based methods (MSP, PSQ, MS-MLPA, MS-PCR), IHC	A: B: C: DNA based methods D:	A: glioblastoma B: C: D:	A: glioblastoma of the elderly B: C: glioblastoma (all patients except elderly) D:	[46, 47, 48, 49, 50, 51, 52, 53, 54]
*MYCC* amplification	medulloblastoma (all)	FISH	A: B: C: FISH D:	A: B: medulloblastoma (all) C: D:	A: B: C: D: medulloblastoma (all)	[55, 56, 57, 58, 59, 60]
*MYCN* amplification	medulloblastoma (clinical high risik group (one or more): large cell anaplastic, myc amplification, metastases; age > 3 years; SHH activation)	FISH	A: B: C: FISH D:	A: B: C: D: medulloblastoma (high risk group)	A: B: C: D: medulloblastoma (high risk group)	[55, 58, 59, 60, 61]
β-catenin mutation	medulloblastoma	IHC in combination with sequencing	A: B: C: IHC in combination with sequencing D:	A: B: medulloblastoma C: D:	A: B: C: D: medulloblastoma	[62, 63, 64, 65, 66, 67, 68]

IDH = isocitrate dehydrogenase; IHC = immunhistochemistry; FISH = fluorescence in situ hybridization; MSP = methylation-specific polymerase chain reaction; PSQ = pyrosequencing; MS-MLPA = methylation-specific multiplex ligation-dependent probe amplification; PCR = polymerase chain reaction; MPLA = multiplex ligation-dependent probe amplification; aCGH = array comparative genomic hybridization; MS = methylation-specific; LOH = PCR based loss of heterozygosity analysis.

## References

[1] Biomarkers and surrogate endpoints: preferred definitions and conceptual framework. Clin Pharmacol Ther. 2001; 69: 89–95 CrossRef 11240971 10.1067/mcp.2001.113989

[2] Gutman S Kessler LG The US Food and Drug Administration perspective on cancer biomarker development. Nat Rev Cancer. 2006; 6: 565–571 CrossRef 16794639 10.1038/nrc1911

[3] Hinestrosa MC Dickersin K Klein P Mayer M Noss K Slamon D Sledge G Visco FM Shaping the future of biomarker research in breast cancer to ensure clinical relevance. Nat Rev Cancer. 2007; 7: 309–315 CrossRef 17384585 10.1038/nrc2113

[4] Hainfellner JA Heinzl H Neuropathological biomarker candidates in brain tumors: key issues for translational efficiency. Clin Neuropathol. 2010; 29: 41–54 20040333 10.5414/npp29041

[5] McDonald KL Aw G Kleihues P Role of biomarkers in the clinical management of glioblastomas: what are the barriers and how can we overcome them? Frontiers in neurology. 2012; 3: 188 23346075 10.3389/fneur.2012.00188 PMC3548232

[6] Holdhoff M Ye X Blakeley JO Blair L Burger PC Grossman SA Diaz LA Use of personalized molecular biomarkers in the clinical care of adults with glioblastomas. J Neurooncol. 2012; 110: 279–285 CrossRef 22930388 10.1007/s11060-012-0968-3

[7] Preusser M Capper D Hartmann C IDH testing in diagnostic neuropathology: review and practical guideline article invited by the Euro-CNS research committee. Clin Neuropathol. 2011; 30: 217–230 21955925 10.5414/np300422

[8] Preusser M Wöhrer A Stary S Höftberger R Streubel B Hainfellner JA Value and limitations of immunohistochemistry and gene sequencing for detection of the IDH1-R132H mutation in diffuse glioma biopsy specimens. J Neuropathol Exp Neurol. 2011; 70: 715–723 CrossRef 21760534 10.1097/NEN.0b013e31822713f0

[9] Capper D Sahm F Hartmann C Meyermann R von Deimling A Schittenhelm J Application of mutant IDH1 antibody to differentiate diffuse glioma from nonneoplastic central nervous system lesions and therapy-induced changes. Am J Surg Pathol. 2010; 34: 1199–1204 CrossRef 20661018 10.1097/PAS.0b013e3181e7740d

[10] Capper D Weissert S Balss J Habel A Meyer J Jäger D Ackermann U Tessmer C Korshunov A Zentgraf H Hartmann C von Deimling A Characterization of R132H mutation-specific IDH1 antibody binding in brain tumors. Brain Pathol. 2010; 20: 245–254 CrossRef 19903171 10.1111/j.1750-3639.2009.00352.x PMC8094636

[11] Hartmann C Meyer J Balss J Capper D Mueller W Christians A Felsberg J Wolter M Mawrin C Wick W Weller M Herold-Mende C Unterberg A Jeuken JW Wesseling P Reifenberger G von Deimling A Type and frequency of IDH1 and IDH2 mutations are related to astrocytic and oligodendroglial differentiation and age: a study of 1,010 diffuse gliomas. Acta Neuropathol. 2009; 118: 469–474 CrossRef 19554337 10.1007/s00401-009-0561-9

[12] van den Bent MJ Hartmann C Preusser M Ströbel T Dubbink HJ Kros JM von Deimling A Boisselier B Sanson M Halling KC Diefes KL Aldape K Giannini C Interlaboratory comparison of IDH mutation detection. J Neurooncol. 2013; 112: 173–178 CrossRef 23358936 10.1007/s11060-013-1056-z

[13] Alexander BM Mehta MP Role of isocitrate dehydrogenase in glioma. Expert Rev Neurother. 2011; 11: 1399–1409 CrossRef 21955197 10.1586/ern.11.134

[14] van den Bent MJ Brandes AA Taphoorn MJ Adjuvant Procarbazine, Lomustine, and Vincristine Chemotherapy in Newly Diagnosed Anaplastic Oligodendroglioma: Long-Term Follow-Up of EORTC Brain Tumor Group Study 26951. J Clin Oncol. 2013; 31: 344–350 23071237 10.1200/JCO.2012.43.2229

[15] Hartmann C Hentschel B Wick W Capper D Felsberg J Simon M Westphal M Schackert G Meyermann R Pietsch T Reifenberger G Weller M Loeffler M von Deimling A Patients with IDH1 wild type anaplastic astrocytomas exhibit worse prognosis than IDH1-mutated glioblastomas, and IDH1 mutation status accounts for the unfavorable prognostic effect of higher age: implications for classification of gliomas. Acta Neuropathol. 2010; 120: 707–718 CrossRef 21088844 10.1007/s00401-010-0781-z

[16] SongTao Q Lei Y Si G YanQing D HuiXia H XueLin Z LanXiao W Fei Y IDH mutations predict longer survival and response to temozolomide in secondary glioblastoma. Cancer Sci. 2012; 103: 269–273 CrossRef 22034964 10.1111/j.1349-7006.2011.02134.x

[17] Qi ST Yu L Lu YT Ou YH Li ZY Wu LX Yao F IDH mutations occur frequently in Chinese glioma patients and predict longer survival but not response to concomitant chemoradiotherapy in anaplastic gliomas. Oncol Rep. 2011; 26: 1479–1485 21874255 10.3892/or.2011.1428

[18] Houillier C Wang X Kaloshi G Mokhtari K Guillevin R Laffaire J Paris S Boisselier B Idbaih A Laigle-Donadey F Hoang-Xuan K Sanson M Delattre JY IDH1 or IDH2 mutations predict longer survival and response to temozolomide in low-grade gliomas. Neurology. 2010; 75: 1560–1566 CrossRef 20975057 10.1212/WNL.0b013e3181f96282

[19] Woehrer A Sander P Haberler C Kern S Maier H Preusser M Hartmann C Kros JM Hainfellner JA FISH-based detection of 1p 19q codeletion in oligodendroglial tumors: procedures and protocols for neuropathological practice – a publication under the auspices of the Research Committee of the European Confederation of Neuropathological Societies (Euro-CNS). Clin Neuropathol. 2011; 30: 47–55 21329613 10.5414/npp30047

[20] Cairncross G Wang M Shaw E Phase III Trial of Chemoradiotherapy for Anaplastic Oligodendroglioma: Long-Term Results of RTOG 9402. J Clin Oncol. 2012.; 10.1200/JCO.2012.43.2674 PMC3732012 23071247

[21] Hsu CY Ho DM Yang CF Chiang H Interobserver reproducibility of MIB-1 labeling index in astrocytic tumors using different counting methods. Mod Pathol. 2003; 16: 951–957 CrossRef 13679460 10.1097/01.MP.0000084631.64279.BC

[22] Prayson RA Castilla EA Hembury TA Liu W Noga CM Prok AL Interobserver variability in determining MIB-1 labeling indices in oligodendrogliomas. Ann Diagn Pathol. 2003; 7: 9–13 CrossRef 12616468 10.1053/adpa.2003.50001

[23] Grzybicki DM Liu Y Moore SA Brown HG Silverman JF D’Amico F Raab SS Interobserver variability associated with the MIB-1 labeling index: high levels suggest limited prognostic usefulness for patients with primary brain tumors. Cancer. 2001; 92: 2720–2726 CrossRef 11745208 10.1002/1097-0142(20011115)92:10<2720::aid-cncr1626>3.0.co;2-z

[24] Preusser M Hoeftberger R Woehrer A Gelpi E Kouwenhoven M Kros JM Sanson M Idbaih A Brandes AA Heinzl H Gorlia T Hainfellner JA van den Bent M Prognostic value of Ki67 index in anaplastic oligodendroglial tumours – a translational study of the European Organization for Research and Treatment of Cancer Brain Tumor Group. Histopathology. 2012; 60: 885–894 CrossRef 22335622 10.1111/j.1365-2559.2011.04134.x

[25] Preusser M Heinzl H Gelpi E Höftberger R Fischer I Pipp I Milenkovic I Wöhrer A Popovici F Wolfsberger S Hainfellner JA Ki67 index in intracranial ependymoma: a promising histopathological candidate biomarker. Histopathology. 2008; 53: 39–47 CrossRef 18613924 10.1111/j.1365-2559.2008.03065.x

[26] Mengel M von Wasielewski R Wiese B Rüdiger T Müller-Hermelink HK Kreipe H Inter-laboratory and inter-observer reproducibility of immunohistochemical assessment of the Ki-67 labelling index in a large multi-centre trial. J Pathol. 2002; 198: 292–299 CrossRef 12375261 10.1002/path.1218

[27] Kuncova K Janda A Kasal P Zamecnik J Immunohistochemical prognostic markers in intracranial ependymomas: systematic review and meta-analysis. Pathol Oncol Res. 2009; 15: 605–614 CrossRef 19301151 10.1007/s12253-009-9160-2

[28] Kurt E Zheng PP Hop WC van der Weiden M Bol M van den Bent MJ Avezaat CJ Kros JM Identification of relevant prognostic histopathologic features in 69 intracranial ependymomas, excluding myxopapillary ependymomas and subependymomas. Cancer. 2006; 106: 388–395 CrossRef 16342252 10.1002/cncr.21608

[29] Wolfsberger S Fischer I Höftberger R Birner P Slavc I Dieckmann K Czech T Budka H Hainfellner J Ki-67 immunolabeling index is an accurate predictor of outcome in patients with intracranial ependymoma. Am J Surg Pathol. 2004; 28: 914–920 CrossRef 15223962 10.1097/00000478-200407000-00011

[30] Widhalm G Wolfsberger S Preusser M Fischer I Woehrer A Wunderer J Hainfellner JA Knosp E Residual nonfunctioning pituitary adenomas: prognostic value of MIB-1 labeling index for tumor progression. J Neurosurg. 2009; 111: 563–571 CrossRef 18991501 10.3171/2008.4.17517

[31] Rishi A Sharma MC Sarkar C Jain D Singh M Mahapatra AK Mehta VS Das TK A clinicopathological and immunohistochemical study of clinically non-functioning pituitary adenomas: a single institutional experience. Neurol India. 2010; 58: 418–423 CrossRef 20644271 10.4103/0028-3886.66336

[32] Chacko G Chacko AG Kovacs K Scheithauer BW Mani S Muliyil JP Seshadri MS The clinical significance of MIB-1 labeling index in pituitary adenomas. Pituitary. 2010; 13: 337–344 CrossRef 20640601 10.1007/s11102-010-0242-7

[33] Uematsu M Ohsawa I Aokage T Nishimaki K Matsumoto K Takahashi H Asoh S Teramoto A Ohta S Prognostic significance of the immunohistochemical index of survivin in glioma: a comparative study with the MIB-1 index. J Neurooncol. 2005; 72: 231–238 CrossRef 15937645 10.1007/s11060-004-2353-3

[34] Torp SH Diagnostic and prognostic role of Ki67 immunostaining in human astrocytomas using four different antibodies. Clin Neuropathol. 2002; 21: 252–257 12489673

[35] Rodríguez-Pereira C Suárez-Peñaranda JM Vázquez-Salvado M Sobrido MJ Abraldes M Barros F Forteza J Value of MIB-1 labelling index (LI) in gliomas and its correlation with other prognostic factors. A clinicopathologic study. J Neurosurg Sci. 2000; 44: 203–209 Discussion 209-210. 11327289

[36] Vaquero J Zurita M Morales C Oya S Coca S Prognostic significance of endothelial surface score and MIB-1 labeling index in glioblastoma. J Neurooncol. 2000; 46: 11–16 CrossRef 10896201 10.1023/a:1006347919565

[37] Heesters MA Koudstaal J Go KG Molenaar WM Analysis of proliferation and apoptosis in brain gliomas: prognostic and clinical value. J Neurooncol. 1999; 44: 255–266 CrossRef 10720205 10.1023/a:1006398613605

[38] Litofsky NS Mix TC Baker SP Recht LD Smith TW Ki-67 (clone MIB-1) proliferation index in recurrent glial neoplasms: no prognostic significance. Surg Neurol. 1998; 50: 579–585 CrossRef 9870820 10.1016/s0090-3019(97)00312-1

[39] Oya S Kawai K Nakatomi H Saito N Significance of Simpson grading system in modern meningioma surgery: integration of the grade with MIB-1 labeling index as a key to predict the recurrence of WHO Grade I meningiomas. J Neurosurg. 2012; 117: 121–128 CrossRef 22559847 10.3171/2012.3.JNS111945

[40] Abry E Thomassen IO Salvesen OO Torp SH The significance of Ki-67/MIB-1 labeling index in human meningiomas: a literature study. Pathol Res Pract. 2010; 206: 810–815 CrossRef 20951502 10.1016/j.prp.2010.09.002

[41] Torp SH Lindboe CF Grønberg BH Lydersen S Sundstrøm S Prognostic significance of Ki-67/MIB-1 proliferation index in meningiomas. Clin Neuropathol. 2005; 24: 170–174 16033133

[42] Roser F Samii M Ostertag H Bellinzona M The Ki-67 proliferation antigen in meningiomas. Experience in 600 cases. Acta Neurochir (Wien). 2004; 146: 37–44 Discussion 44. CrossRef 14740263 10.1007/s00701-003-0173-4

[43] Das P Puri T Suri V Medulloblastomas: a correlative study of MIB-1 proliferation index along with expression of c-Myc, ERBB2, and anti-apoptotic proteins along with histological typing and clinical outcome. Child’s nervous system: ChNS: official journal of the International Society for Pediatric Neurosurgery. 2009; 25: 825–835 10.1007/s00381-009-0884-9 19444455

[44] Ferrari AF Araújo MB Aguiar PH Plese JP Medulloblastoma: evaluation of proliferative index by monoclonal antibody Mib-1, its prognostic correlation and therapeutic implications. Arq Neuropsiquiatr. 2003; 61: 547–551 CrossRef 14513155 10.1590/s0004-282x2003000400004

[45] Hegi ME Janzer RC Lambiv WL Gorlia T Kouwenhoven MC Hartmann C von Deimling A Martinet D Besuchet Schmutz N Diserens AC Hamou MF Bady P Weller M van den Bent MJ Mason WP Mirimanoff RO Stupp R Mokhtari K Wesseling P Presence of an oligodendroglioma-like component in newly diagnosed glioblastoma identifies a pathogenetically heterogeneous subgroup and lacks prognostic value: central pathology review of the EORTC_26981/NCIC_CE.3 trial. Acta Neuropathol. 2012; 123: 841–852 CrossRef 22249618 10.1007/s00401-011-0938-4

[46] Berghoff AS Preusser M Clinical neuropathology practice guide 06-2012: MGMT testing in elderly glioblastoma patients – yes, but how? Clin Neuropathol. 2012; 31: 405–408 23083460 10.5414/NP300576 PMC3663470

[47] Weller M Stupp R Reifenberger G Brandes AA van den Bent MJ Wick W Hegi ME MGMT promoter methylation in malignant gliomas: ready for personalized medicine? Nat Rev Neurol. 2010; 6: 39–51 CrossRef 19997073 10.1038/nrneurol.2009.197

[48] Preusser M Charles Janzer R Felsberg J Reifenberger G Hamou MF Diserens AC Stupp R Gorlia T Marosi C Heinzl H Hainfellner JA Hegi M Anti-O6-methylguanine-methyltransferase (MGMT) immunohistochemistry in glioblastoma multiforme: observer variability and lack of association with patient survival impede its use as clinical biomarker. Brain Pathol. 2008; 18: 520–532 18400046 10.1111/j.1750-3639.2008.00153.x PMC8095504

[49] Jeuken JW Cornelissen SJ Vriezen M MS-MLPA: an attractive alternative laboratory assay for robust, reliable, and semiquantitative detection of MGMT promoter hypermethylation in gliomas. Laboratory investigation; a journal of technical methods and pathology. 2007; 87: 1055–1065 10.1038/labinvest.3700664 17700563

[50] Preusser M MGMT analysis at DNA, RNA and protein levels in glioblastoma tissue. Histol Histopathol. 2009; 24: 511–518 19224454 10.14670/HH-24.511

[51] Hegi ME Diserens AC Gorlia T Hamou MF de Tribolet N Weller M Kros JM Hainfellner JA Mason W Mariani L Bromberg JE Hau P Mirimanoff RO Cairncross JG Janzer RC Stupp R MGMT gene silencing and benefit from temozolomide in glioblastoma. N Engl J Med. 2005; 352: 997–1003 CrossRef 15758010 10.1056/NEJMoa043331

[52] Gilbert MR Wang M Aldape KD RTOG 0525: A randomized phase III trial comparing standard adjuvant temozolomide (TMZ) with a dose-dense (dd) schedule in newly diagnosed glioblastoma (GBM). J Clin Oncol. 2011; 29 :

[53] Wick W Platten M Meisner C Felsberg J Tabatabai G Simon M Nikkhah G Papsdorf K Steinbach JP Sabel M Combs SE Vesper J Braun C Meixensberger J Ketter R Mayer-Steinacker R Reifenberger G Weller M Temozolomide chemotherapy alone versus radiotherapy alone for malignant astrocytoma in the elderly: the NOA-08 randomised, phase 3 trial. Lancet Oncol. 2012; 13: 707–715 CrossRef 22578793 10.1016/S1470-2045(12)70164-X

[54] Malmström A Grønberg BH Marosi C Stupp R Frappaz D Schultz H Abacioglu U Tavelin B Lhermitte B Hegi ME Rosell J Henriksson R Temozolomide versus standard 6-week radiotherapy versus hypofractionated radiotherapy in patients older than 60 years with glioblastoma: the Nordic randomised, phase 3 trial. Lancet Oncol. 2012; 13: 916–926 CrossRef 22877848 10.1016/S1470-2045(12)70265-6

[55] Pfister S Remke M Benner A Outcome prediction in pediatric medulloblastoma based on DNA copy-number aberrations of chromosomes 6q and 17q and the MYC and MYCN loci. ournal of clinical oncology: official journal of the American Society of Clinical Oncology. 2009; 27: 1627–1636 10.1200/JCO.2008.17.9432 19255330

[56] Aldosari N Bigner SH Burger PC Becker L Kepner JL Friedman HS McLendon RE MYCC and MYCN oncogene amplification in medulloblastoma. A fluorescence in situ hybridization study on paraffin sections from the Children’s Oncology Group. Arch Pathol Lab Med. 2002; 126: 540–544 11958658 10.5858/2002-126-0540-MAMOAI

[57] Lamont JM McManamy CS Pearson AD Combined histopathological and molecular cytogenetic stratification of medulloblastoma patients. Clinical cancer research: an official journal of the American Association for Cancer Research. 2004; 10: 5482–5493 15328187 10.1158/1078-0432.CCR-03-0721

[58] Ryan SL Schwalbe EC Cole M Lu Y Lusher ME Megahed H O’Toole K Nicholson SL Bognar L Garami M Hauser P Korshunov A Pfister SM Williamson D Taylor RE Ellison DW Bailey S Clifford SC MYC family amplification and clinical risk-factors interact to predict an extremely poor prognosis in childhood medulloblastoma. Acta Neuropathol. 2012; 123: 501–513 CrossRef 22139329 10.1007/s00401-011-0923-y

[59] Ellison DW Kocak M Dalton J Definition of disease-risk stratification groups in childhood medulloblastoma using combined clinical, pathologic, and molecular variables. Journal of clinical oncology: official journal of the American Society of Clinical Oncology. 2011; 29: 10.1200/JCO.2010.30.2810 PMC3525837 20921458

[60] von Hoff K Hartmann W von Bueren AO Gerber NU Grotzer MA Pietsch T Rutkowski S Large cell/anaplastic medulloblastoma: outcome according to myc status, histopathological, and clinical risk factors. Pediatr Blood Cancer. 2010; 54: 369–376 CrossRef 19908297 10.1002/pbc.22339

[61] Korshunov A Remke M Kool M Hielscher T Northcott PA Williamson D Pfaff E Witt H Jones DT Ryzhova M Cho YJ Wittmann A Benner A Weiss WA von Deimling A Scheurlen W Kulozik AE Clifford SC Peter Collins V Westermann F Biological and clinical heterogeneity of MYCN-amplified medulloblastoma. Acta Neuropathol. 2012; 123: 515–527 CrossRef 22160402 10.1007/s00401-011-0918-8

[62] Eberhart CG Tihan T Burger PC Nuclear localization and mutation of beta-catenin in medulloblastomas. J Neuropathol Exp Neurol. 2000; 59: 333–337 10759189 10.1093/jnen/59.4.333

[63] Clifford SC Lusher ME Lindsey JC Langdon JA Gilbertson RJ Straughton D Ellison DW Wnt/Wingless pathway activation and chromosome 6 loss characterize a distinct molecular sub-group of medulloblastomas associated with a favorable prognosis. Cell Cycle. 2006; 5: 2666–2670 CrossRef 17172831 10.4161/cc.5.22.3446

[64] Fattet S Haberler C Legoix P Varlet P Lellouch-Tubiana A Lair S Manie E Raquin MA Bours D Carpentier S Barillot E Grill J Doz F Puget S Janoueix-Lerosey I Delattre O Beta-catenin status in paediatric medulloblastomas: correlation of immunohistochemical expression with mutational status, genetic profiles, and clinical characteristics. J Pathol. 2009; 218: 86–94 CrossRef 19197950 10.1002/path.2514

[65] Kool M Korshunov A Remke M Jones DT Schlanstein M Northcott PA Cho YJ Koster J Schouten-van Meeteren A van Vuurden D Clifford SC Pietsch T von Bueren AO Rutkowski S McCabe M Collins VP Bäcklund ML Haberler C Bourdeaut F Delattre O Molecular subgroups of medulloblastoma: an international meta-analysis of transcriptome, genetic aberrations, and clinical data of WNT, SHH, Group 3, and Group 4 medulloblastomas. Acta Neuropathol. 2012; 123: 473–484 CrossRef 22358457 10.1007/s00401-012-0958-8 PMC3306778

[66] Ellison DW Onilude OE Lindsey JC beta-Catenin status predicts a favorable outcome in childhood medulloblastoma: the United Kingdom Children’s Cancer Study Group Brain Tumour Committee. Journal of clinical oncology: official journal of the American Society of Clinical Oncology. 2005; 23: 7951-7957 16258095 10.1200/JCO.2005.01.5479

[67] Ellison DW Dalton J Kocak M Nicholson SL Fraga C Neale G Kenney AM Brat DJ Perry A Yong WH Taylor RE Bailey S Clifford SC Gilbertson RJ Medulloblastoma: clinicopathological correlates of SHH, WNT, and non-SHH/WNT molecular subgroups. Acta Neuropathol. 2011; 121: 381–396 CrossRef 21267586 10.1007/s00401-011-0800-8 PMC3519926

[68] Koch A Waha A Tonn JC et al. Somatic mutations of WNT/wingless signaling pathway components in primitive neuroectodermal tumors. International journal of cancer. Journal international du cancer. 2001; 93: 445-449 11433413 10.1002/ijc.1342

[69] Preusser M Bent Mv Clinical Neuropathology Practice News 2-2013: immunohistochemistry pins IDH in glioma - molecular testing procedures under scrutiny. Clin Neuropathol. 2013; 32: 82–83 23442355 10.5414/NP300622 PMC3663465

